# A Tooth Decaying in the Appendix: An Unusual Cause of Appendicitis

**DOI:** 10.7759/cureus.24086

**Published:** 2022-04-12

**Authors:** Zachary J Brennan, Grace Young, Kyle Packer

**Affiliations:** 1 Surgery, Michigan State University College of Osteopathic Medicine, East Lansing, USA; 2 Surgery, A.T. Still University-Kirksville College of Osteopathic Medicine, Kirksville, USA; 3 General Surgery, Brookwood Baptist Health, Jasper, USA

**Keywords:** robotic surgery education, robotic surgery procedures, laparotomy in appendicitis, diagnosis of acute appendicitis, atypical appendicitis

## Abstract

Appendicitis is a very common indication for surgery, although in recent years uncomplicated cases have often been managed with antibiotics. In this case, we discuss a patient who presented to the emergency department with a case of seemingly uncomplicated acute appendicitis. Physical exam, history, and imaging indicated that this was due to an ingested foreign body, specifically a dental crown, that had impacted the appendix. In cases of ingested foreign bodies, antibiotics are not an appropriate treatment for appendicitis and all cases should be treated surgically if the patient will tolerate surgery. A thorough history and physical exam, as well as imaging when indicated, can assist in the assessment of such patients.

## Introduction

Appendicitis is one of the classic indications for surgery. Appendicitis is inflammation of the appendix, usually caused by blockage of the lumen of the appendix, and when left untreated can lead to serious conditions like peritonitis [1-3]. Among the most common surgical procedures performed in the United States annually, appendectomy is at the top of the list with more than 300,000 operations performed each year [1]. While a laparoscopic approach is still the most common form of operative management, robotic-assisted surgery is a growing method of removing the appendix [2]. In cases of acute appendicitis, prompt removal can prevent more serious complications including but not limited to infection, abscess, peritonitis, and bowel obstruction [2]. Clinical evaluation and management are the mainstays of diagnosis, though imaging can be of assistance. Imaging is essential in stratifying cases of acute appendicitis as simple or complex based on whether they are non-perforated (simple) or perforated/gangrenous (complex) [3].

While some simple cases of appendicitis may respond to non-operative management (treated exclusively with antibiotics), especially those where patients present with multiple days of symptoms, appendicitis caused by a foreign body requires prompt surgical intervention [4]. Ingested foreign bodies that obstruct the appendiceal lumen are very likely to cause appendicitis, and these cases frequently result in complications such as rupture and infection [4-6].

Here, we present a case of a patient who was determined to have an unusual case of appendicitis caused by an ingested foreign body to highlight the importance of a complete history and physical exam for suspected appendicitis and the critical role imaging plays as a diagnostic tool when faced with a possible foreign body cause of appendicitis.

## Case presentation

This case involved a 51-year-old male patient who presented with acute onset right lower quadrant pain with nausea and dry heaving. The patient stated that the pain developed over several days and eventually localized to the right lower quadrant. The patient stated that the pain was progressively worsening, sharp in nature, and associated with nausea and dry heaving at the time of presentation. Medical and surgical history was non-contributory, but dental history revealed that the patient recently had dental crowns placed on six of his teeth, and most of the crowns had either fallen out or he had coughed them up. The patient stated that he could have swallowed one of the crowns. There were no physical exam findings of acute abdomen or findings specific to appendicitis; however, suspicion was high for appendicitis based on patient history. In the emergency department, the patient was hemodynamically stable with a blood pressure of 125/68 mmHg, temperature of 98.2F, pulse of 73, and a pulse oximeter reading of 97%. White blood cell count was elevated at 20.0 x10^9^/L. 

Due to suspected appendicitis, the patient was sent for both a plain abdominal x-ray and computed tomography scan. Imaging revealed a 1 x 0.9 cm metallic density in the appendix near its origin in the cecum, as well as inflammatory findings consistent with acute appendicitis. Regional right lower quadrant ileus was also noted on imaging.

**Figure 1:**
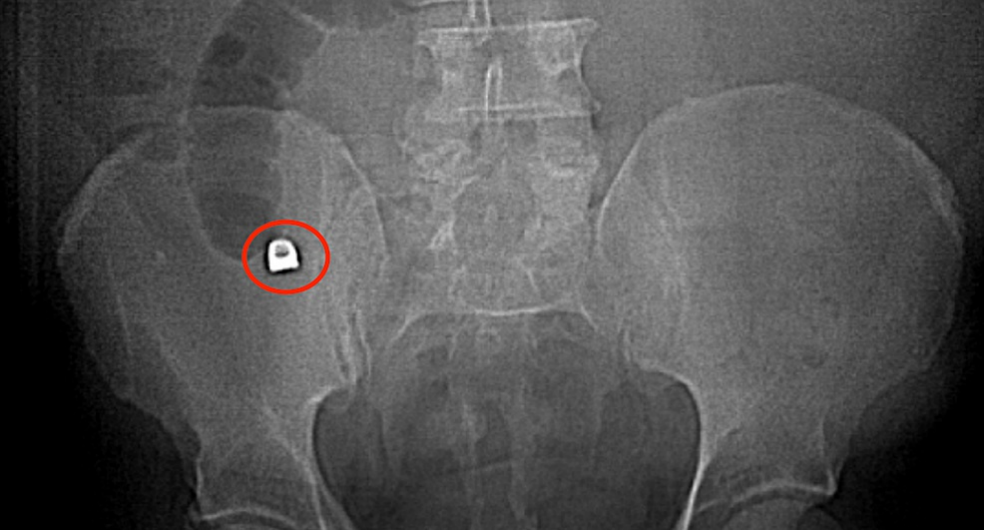
Plain film showing metallic object in appendix

**Figure 2:**
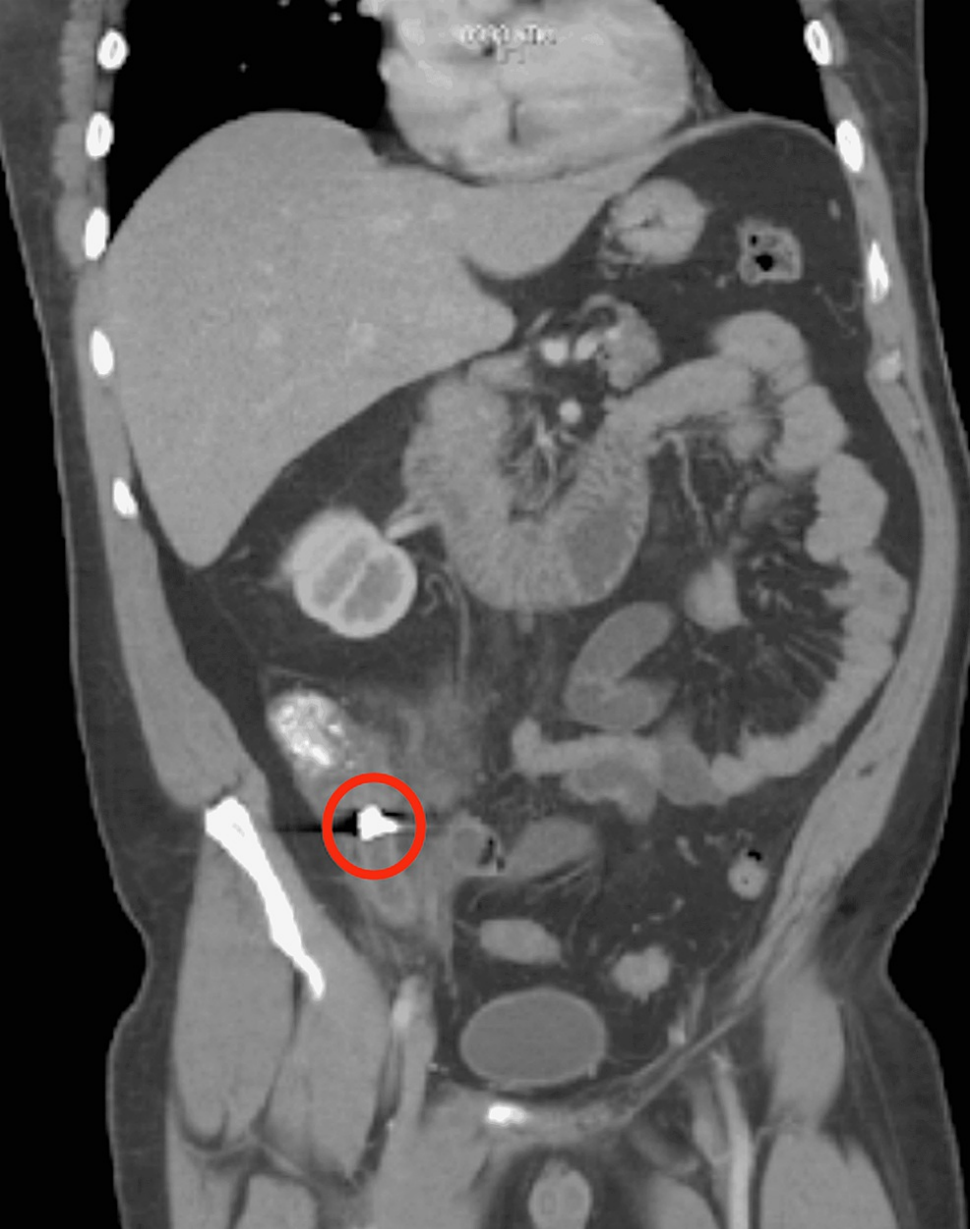
Computed tomography scan showing metallic object in appendix

The patient was subsequently diagnosed with acute appendicitis based on clinical picture and imaging, and a robotic-assisted surgery was planned for appendectomy. The patient was started on appropriate prophylactic preoperative antibiotics.

A robotic-assisted appendectomy was completed without complication. Upon examining the contents of the endo-catch bag, it was discovered that the patient’s appendicitis was caused by a dental crown that had been swallowed by the patient previously. The patient recovered without complications after the procedure.

## Discussion

Foreign objects causing appendicitis are a somewhat rare presentation, accounting for approximately 0.005% of total cases per year in the United States [4]. This case was a straightforward appendicitis case which would normally not warrant much attention, except for the rarity of foreign objects causing appendicitis. More specifically, the rarity of appendicitis is caused by a dental crown that a patient mistakenly swallowed. This patient admitted to coughing up two of his dental crowns but stated that one was accidentally swallowed. Imaging and good clinical history taking were of vital importance in determining the cause of appendicitis, highlighting these important tools even in a case as common as appendicitis [6]. An extensive review of one hundred years of ingested foreign bodies causing appendicitis suggests these cases often cause perforation and are almost always radiopaque, again highlighting the utility of imaging in these cases [7].

There has been a debate in the literature recently about the use of appendectomy compared to antibiotics for appendicitis, especially in cases that are initially thought to be uncomplicated. Though appendectomy is still considered a first-line treatment, interval appendectomy is sometimes considered for delayed presentation [8]. There are multiple well-described risk factors for those who initially undergo non-operative management with only antibiotics to eventually require an appendectomy. Two of the top factors leading to the necessity of operative management include obstruction of the lumen of the appendix by a fecalith and a diameter of the appendix greater than 1 cm on imaging [9]. Some studies have argued that antibiotic management is appropriate and evolution of treatment in uncomplicated appendicitis [10,11]. Of cases initially treated with antibiotics alone, the rate of recurrence eventually requiring appendectomy is significant, with 29% requiring surgical management within 90 days; 27.3% within one year; and 39.1% within five years [11]. Although unusual cases of appendicitis are uncommon, because some cases of appendicitis have rare etiologies, histopathologic examination of appendix samples after surgery is important [12,13].

Despite studies showing antibiotics can be used for uncomplicated appendicitis, cases associated with an obstruction or a swallowed foreign body are not amenable to non-operative management and should instead be scheduled for urgent or emergent surgery depending on the clinical picture; this is true even when the clinical picture may indicate uncomplicated appendicitis. Imaging is very useful in such cases, and a thorough history and physical examination are important in order to establish the possibility of a foreign body being the cause of appendicitis.

## Conclusions

While appendicitis presenting after several days of symptoms is sometimes managed with an interval appendectomy, antibiotic usage is a growing modality of non-operative management. In the event of appendicitis secondary to luminal obstruction with a foreign body, an urgent appendectomy is indicated. This case highlights the importance of thorough history taking in patients presenting with acute or chronic appendicitis in order to determine if there is any sort of inciting event that may have caused appendicitis. This is especially true if an ingested foreign body becomes trapped in the lumen of the appendix. Imaging, while often unnecessary in the initial diagnosis of acute appendicitis, can also be vital in showing a foreign body in the appendix, as with this patient.

## References

[1] Skendelas JP, Alemany VS, Au V, Rao D, McNelis J, Kim PK (2021). Appendiceal adenocarcinoma found by surgery for acute appendicitis is associated with older age. BMC Surg.

[2] Quilici PJ, Wolberg H, McConnell N (2022). Operating costs, fiscal impact, value analysis and guidance for the routine use of robotic technology in abdominal surgical procedures. Surg Endosc.

[3] Bhangu A, Søreide K, Di Saverio S, Assarsson JH, Drake FT (2015). Acute appendicitis: modern understanding of pathogenesis, diagnosis, and management. Lancet.

[4] Klingler PJ, Smith SL, Abendstein BJ, Brenner E, Hinder RA (1997). Management of ingested foreign bodies within the appendix: a case report with review of the literature. Am J Gastroenterol.

[5] Balch CM, Silver D (1971). Foreign bodies in the appendix. Report of eight cases and review of the literature. Arch Surg.

[6] Glen P, Ihedioha U, Mackenzie I (2007). An unusual extraction; retrieval of a swallowed crown by appendicectomy. Br Dent J.

[7] Klingler PJ, Seelig MH, DeVault KR, Wetscher GJ, Floch NR, Branton SA, Hinder RA (1998). Ingested foreign bodies within the appendix: a 100-year review of the literature. Dig Dis.

[8] Moris D, Paulson EK, Pappas TN (2021). Diagnosis and management of acute appendicitis in adults: a review. JAMA.

[9] Monsell SE, Voldal EC, Davidson GH (2022). Patient factors associated with appendectomy within 30 days of initiating antibiotic treatment for appendicitis. JAMA Surg.

[10] Barie PS (2021). Non-operative management of appendicitis: evolution, not revolution. Surg Infect (Larchmt).

[11] Grasso CS, Walker LA (2021). Modern management of the appendix: so many options. Surg Clin North Am.

[12] Yilmaz M, Akbulut S, Kutluturk K, Sahin N, Arabaci E, Ara C, Yilmaz S (2013). Unusual histopathological findings in appendectomy specimens from patients with suspected acute appendicitis. World J Gastroenterol.

[13] Akbulut S, Tas M, Sogutcu N (2011). Unusual histopathological findings in appendectomy specimens: a retrospective analysis and literature review. World J Gastroenterol.

